# Report on the occurrence of synanthropic derived form of *Chrysomya megacephala* (Diptera: Calliphoridae) from Royapuram fishing harbour, Chennai, Tamil Nadu, India

**DOI:** 10.3897/BDJ.2.e1111

**Published:** 2014-06-26

**Authors:** Paulchamy Ramaraj, Chellappa Selvakumar, Arumugam Ganesh, Sundaram Janarthanan

**Affiliations:** † Department of Zoology, University of Madras, Chennai, India

**Keywords:** Blowfly, Molecular identification, DNA barcoding, India

## Abstract

The occurrence of dipteran fly, *Chrysomya megacephala* (Fabricius, 1794) is reported for the first time from Royapuram fishing harbour (Chennai), Tamil Nadu, South East India. The fully grown third instar larvae of *Chrysomya megacephala* were collected from decaying fishes near Royapuram fishing harbour. This site is found to be the regular breeding site for *Chrysomya megacephala*. Larvae were reared under laboratory condition and freshly emerged adult flies from pupae were collected and identified by morphological features and molecular tools. Molecular identification through generation of DNA barcoding using mitochondrial COI gene of *Chrysomya megacephala* is appended.

## Introduction

Calliphoridae is a cosmopolitan group of calyptrate flies comprising nearly 1500 recognized species worldwide (De Carvalho and Mello-Patiu 2008). The Indian representatives of this family belong to 63 species under 18 genera (Nandi 2004; Singh and Sidhu 2004, Singh and Sidhu 2007). *Chrysomya* is one of the most significant genera of Calliphoridae with great medical, veterinary and forensic importance. Ten species of the genus *Chrysomya* have been recorded in India (Bharti 2011).

*Chrysomya megacephala* (Fabricius, 1794) is commonly found in cadavers in many parts of the world (Gruner et al. 2007, Sukontason et al. 2007, Wang et al. 2008), and is used in forensic investigations to determine post-mortem intervals (Greenberg 1971, Goff and Odom 1987, Goff et al. 1988, Goff 1992, Centeno et al. 2002). It is a tremendously abundant species, dispersed rapidly throughout the Oriental, Australasian, Afrotropical, Neotropical, Nearctic and Palearctic zoogeographical regions, (Smith 1986, Castro and García 2009), and used as fish bait in northern and south-west India (Roy and Dasgupta 1975, Wall et al. 2001). It is known to breed in human faeces, decomposed meat and fish (Laurence 1981, Laurence 1986) as well as discarded organic materials. This dipteran fly, therfore, serves as a potential vector of many diseases due to its close association with human dwellings. In general, *Chrysomya megacephala* is considered important in medical, veterinary and forensic sciences (Tantawi et al. 1996, De Souza and Linhares 1997, Centeno et al. 2002, Smith 1986, Wells and Kurahashi 1994).

Presently, three forms of *Chrysomya megacephala* are recognized, namely, the normal form (nf), the synanthropic derived form (sdf) and the recently reported feral derived form (fdf) (Bharti and Kurahashi 2009). The normal form is confined to forests of South Pacific Islands (from Bismarck Archipelago to Western Samoa) while synanthropic derived form has spread around the world from Papua New Guinea. The recently reported feral derived form (fdf) in the forests of the Himalaya, India is a morphological intermediate between normal and synanthropic derived forms (Bharti and Kurahashi 2009). Synanthropic derived form of *Chrysomya megacephala* is common around human dwellings enabling direct contact with humans. It occurs on dead fish, sweets, carcasses, human excrement and fruits. The adult flies are vectors of a few infectious diseases of the digestive tract and reported to carry *Morganella* sp. (Enterobacteriales: Enterobacteriaceae), which causes summer diarrhoea (Sinha and Nandi 2004). It is also associated with many microbial pathogens including polio virus. Besides, this fly has been reported as vectors of enteric pathogens in malnourished individuals living under unsanitary conditions (Greenberg 1971). The larvae of *Chrysomya megacephala* are parasitic on semi-dried fish, causing a major problem in the fish industry of Southeast Asia (Esser 1991, Wall et al. 2001). It is a secondary myiasis producer in man and in domestic animals (Sinha and Nandi 2004).

However, the occurrence of the synanthropic derived form of *Chrysomya megacephala* has not been documented in the State of Tamil Nadu, India. In this context, the present study reports for the first time the synanthropic derived form of *Chrysomya megacephala* from Royapuram fishing harbour, Chennai, Tamil Nadu, South East India and provides key characters based on morphological features and molecular analysis.

## Materials and methods

**Collection, rearing and morphological identification**

**Figure 1.**
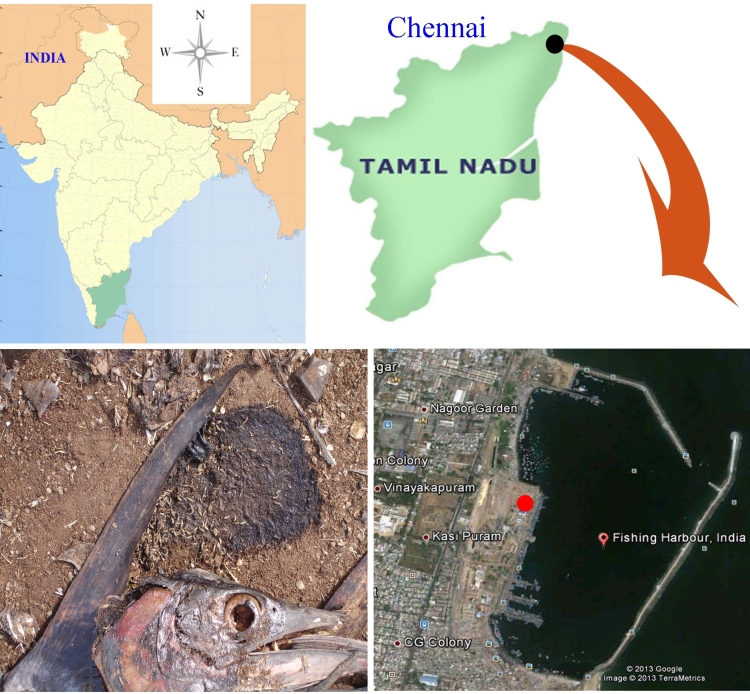
Map showing collection site of Royapuram fishing harbour of North Chennai, Tamil Nadu

A colony of *Chrysomya megacephala* was established from numerous second and third instar larvae collected from decaying fishes of Royapuram fishing harbour in North Chennai, Tamil Nadu, South India (Fig. 1). The colony of *Chrysomya megacephala* used in this study was successfully cultured in the insectarium at the Unit of Entomology, Department of Zoology, University of Madras. Colonies were maintained at 28 °C and R.H. of 80%. Freshly emerged adults from pupae were morphologically identified using the keys provided by Senior-White et al. 1940. Voucher specimens were deposited at Zoological Survey of India, Southern Regional Centre, Chennai, Tamil Nadu, India (I/D 28: 3 male and I/D 29: 3 female).

### Morphological description

The procedure adopted to identify the morphological features and terminology used in the description were based on the previous reports of Sukontason et al. 2008, Siriwattanarungsee et al. 2005, Sinha and Nandi 2004.

### Molecular identification

Genomic DNA was extracted from a single morphologically identified adult male and female species of *Chrysomya megacephala* (sdf) after removing the gut region as per the standard phenol/chloroform extraction protocol (Murry and Thompson 1980).The extracted DNA was quantified using spectrophotometer and diluted to 50ng/µl prior to PCR amplification. The mitochondrial cytochrome-c oxidase subunit I (COI) gene was amplified using the primer pair LCO1490 (5’-GGTCAACAAATCATAAAGATATTGG-3’) and HCO2198 (5’ TAAACTTCAGGGTGACCAAAAAATCA-3’) under standard PCR conditions (Folmer et al. 1994). The amplified PCR product was purified from agarose gel using PCR product purification kit (Qiagen). The purified PCR products were used for sequencing with Big Dye on ABI 3730 automated DNA sequencer. BLAST analysis was performed using these sequences to identify matches from the database.

## Taxon treatments

### Chrysomya megacephala

Fabricius, 1794

AB910389

AB910390

SPLID033-14

SPLID013-13

#### Materials

- **Type status:** Other material. **Occurrence:** catalogNumber: I/D 28: 3 male and I/D 29: 3 female; recordedBy: Dr. S. Janarthanan; individualCount: 25; sex: 12 male, 13 female; disposition: Zoological Survey of India, Southern Regional Centre, Chennai, Tamil Nadu, India; **Taxon:** kingdom: Animalia; phylum: Arthropoda; class: Insecta; order: Diptera; family: Calliforidae; genus: Chrysomya; specificEpithet: megacephala; taxonRank: species; vernacularName: Oriental latrine fly; taxonomicStatus: accepted; **Location:** country: India; stateProvince: Tamil Nadu; municipality: Chennai; verbatimLocality: Royapuram fishing horbour; verbatimElevation: 2 m; verbatimLatitude: 13°07'44.73 N; verbatimLongitude: 80°17'52.70 E; **Identification:** identifiedBy: P. Ramaraj & C. Selvakumar; dateIdentified: 29 Jan 2014; identificationReferences: Senior-White et al., 1940; identificationRemarks: Eye facets of upper two-thirds greately enlarged and sharply demarcated from small facets of lower third; **Event:** samplingProtocol: Hand picking; eventDate: 2013-12-22; **Record Level:** institutionCode: University of Madras, Chennai

#### Diagnosis

**Larva**: Fully mature third instar muscoid-shaped (11 mm length), composed of 12 segments with pointed anterior and blunt posterior end (Fig. 2a). Cephaloskeleton present in anterior end; dorsal cornu longer than ventral cornua; accessory sclerite, parastomal sclerite present; anterodorsal process apparent (Fig. 2b). Anterior spiracle possessed eight to 12 marginal branches (Fig. 2c). Dorsal cuticular spines between the prothorax and mesothorax arranged single, each having one to three dark pointed tips (Fig. 2d). Inner dorsal tubercle moderate between middle dorsal tubercle and outer dorsal tubercle, with latter tubercle largest; a pair of the posterior spiracles clearly visible at the caudal end (Fig. 2e). Peritreme incomplete and heavily pigmented as a dark structure encircling three relatively straight spiracular openings (slits), with middle slits appearing slightly bent inward (Fig. 2f).

##### Pupa

Larger in size than larvae; cylindrical in shape (Fig. 3a). A pair of anterior spiracles located laterally on the prothorax at the anterior tip (Fig. 3b). A pair of posterior spiracles located prominently on the contracted surface of caudal region. Each spiracle more or less an oval shaped peritreme, encircling three straight spiracular slits (Fig. 3c).

##### Adult

**Male** (Fig. 4a): Body length 10-11 mm.

Head: Eye facets of upper two-thirds greatly enlarged and sharply demarked from small facets of lower third (Fig. 4c). Antenna short, postpedicel four times length of pedicel, pedicel dark brown, third brownish; arista dark brown, long plumose; palpi yellowish and slender (Fig. 5a). Thorax: Prostigmatic bristles present; anterior and posterior thoracic spiracles dark brown. Wings: Stem-vein with a row of setulae on upper posterior side; upper surface of lower lobe of squama hairy; upper squama white, partly dark margined; lower squama brown (Fig. 5b). Abdomen: Genital tergites bluish with black hairs; inner forceps elongated, outer forceps reduced (Fig. 5c); anterior paramere wide, slightly curved anteriorly; posterior paramere bifurcated at end; acrophallus wide at end and with a wide stakled projection posteriorly (Fig. 5d).

**Female:** (Fig. 4b). Body length 10-11 mm.

Head: Eyes separated by one-quarter total width of head; facets uniformly small; frons clearly separated; widest part of frons more than width of ocellar triangle; frontal reddish to black, with small hairs on the upper part; frontal bristles short and weak; parafrontalia slightly narrower than width of frons, covered with golden tomentum, but appearing black towards vertex in certain lights; parafacilia yellowish brown with silvery pollen and white hairs (Fig. 4d).

##### Molecular identification and DNA barcoding

The 651 bp mitochondrial cytochrome c oxidase subunit I (COI) sequences of *Chrysomya megacephala* (sdf) were 100% identical with available NCBI database sequences of *Chrysomya megacephala* using BLAST analysis. These mtCOI gene sequences of *Chrysomya megacephala* were submitted to NCBI database (Accession No. AB910389-male; AB910390-female) and DNA barcodes were generated for both the sexes based on their COI sequences using Barcode of Life Database (BOLD system; Process ID SPLID013-13 and SPLID033-14).

#### Distribution

South India – Tamil Nadu (Chennai); Kerala (Calicut); North India - West Bengal and rapidly throughout the continent.

#### Ecology

**Life cycle**: Second and third instar larvae of *Chrysomya megacephala* (sdf) were collected from decaying fishes of Royapuram fishing harbour of North Chennai, Tamil Nadu, South India. Adults emerged in the rearing chamber 7-9 d after pupation. Mating began 2 d after emergence and oviposition occurred at 3-4 d of age and laid egg mass on pieces of spoiled chicken in a squat cup. Each egg mass contained 200-300 eggs. Eggs held at 28 °C hatched on 1 d. Second instars emerged 2 d after hatching, becoming 3rd instar 2 d later, and then pupation occurred 4-6 d.

## Discussion

This study reports for the first time the occurrence of *Chrysomya megacephala* (sdf) in Tamil Nadu, India and provides, therefore, key identification characters of this blowfly based on distinct morphological features of larvae, pupae and adult males and females as well as molecular barcode analysis in the adults of both sexes. *Chrysomya megacephala* also known as the oriental latrine fly (Zumpt 1965), and it is considered one of the most dangerous dipteran vector (Wells 1991) imposing a heavy potential hygienic risk (Maldonado and Centeno 2003). This fly has been expanding its range throughout the world. In India, this species has been reported from north and south-west regions (Roy and Dasgupta 1975, Wall et al. 2001, Nandi 2004, Bharti and Kurahashi 2009, Bharti 2011). The present study is a report on the occurrence of *Chrysomya megacephala* for the first time in south-east India (coastal regions of Tamil Nadu, especially Royapuram coast) in considerable abundance on fish wastes.

## Supplementary Material

XML Treatment for Chrysomya megacephala

## Figures and Tables

**Figure 2a.**
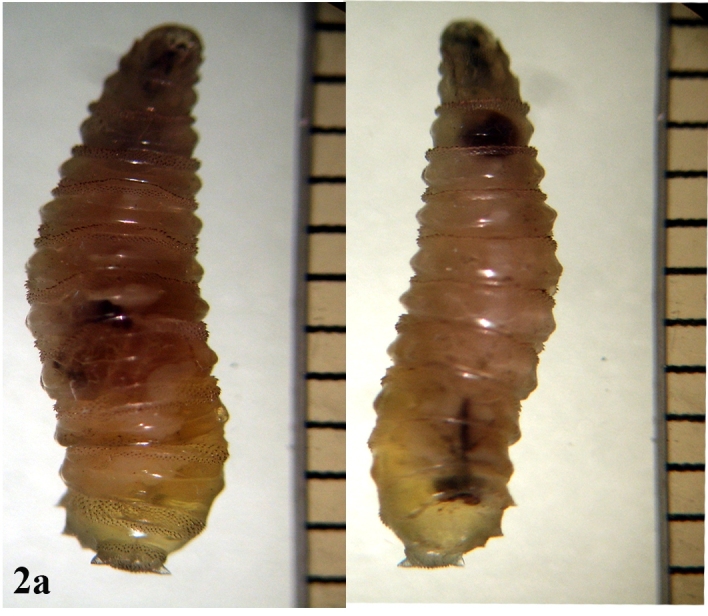
Dorsal view and Ventral view

**Figure 2b.**
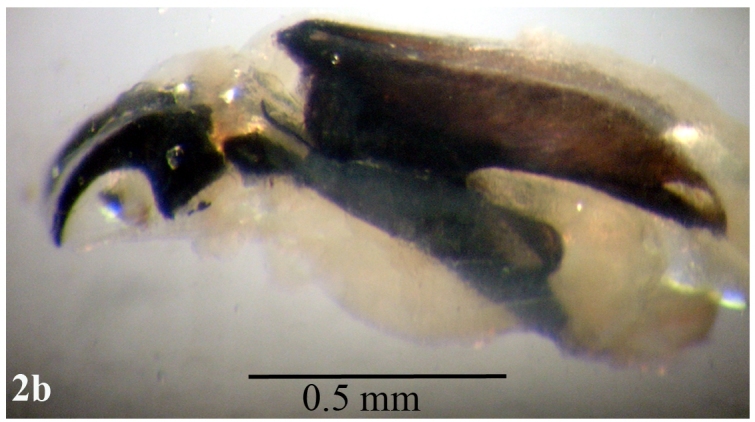
Lateral view of the cephaloskeleton

**Figure 2c.**
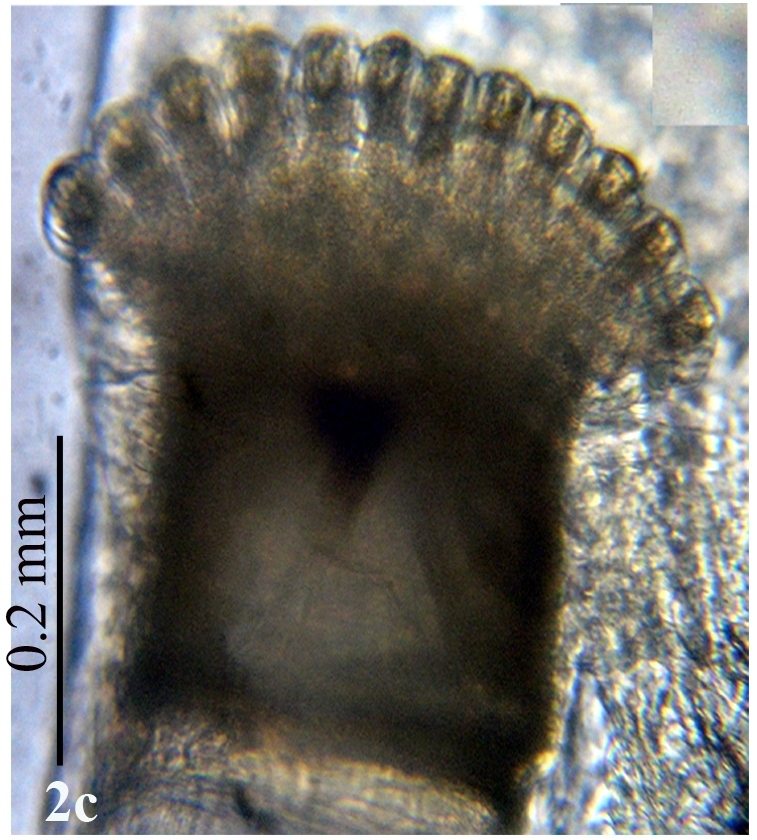
Anterior spiracle

**Figure 2d.**
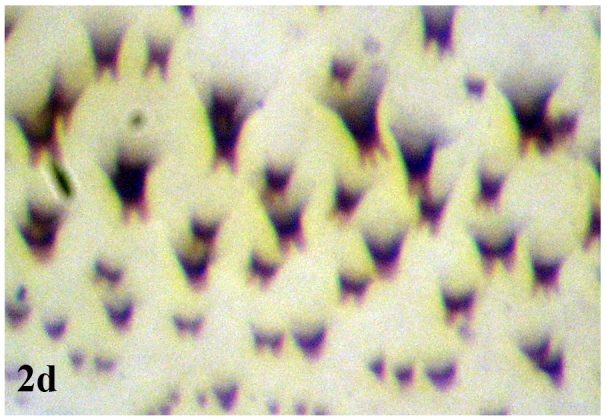
Dorsal cuticular spines

**Figure 2e.**
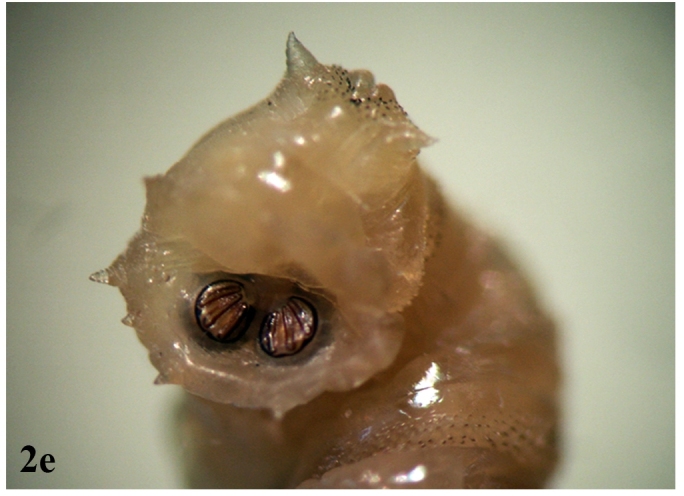
Caudal tubercles and posterior spiracle

**Figure 2f.**
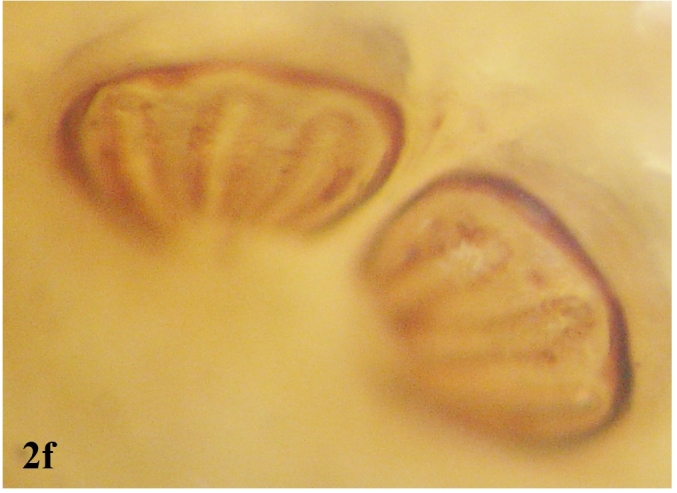
Posterior spiracular openings (slits)

**Figure 3a.**
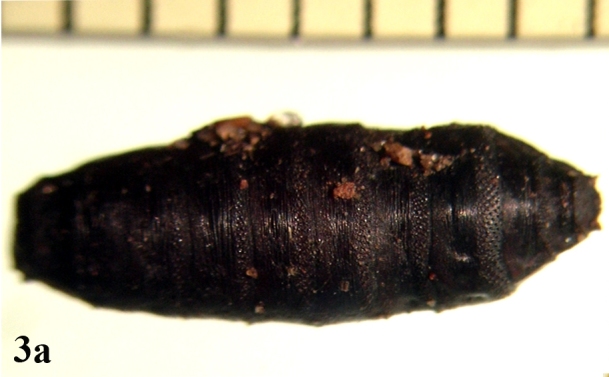
Dorsal view

**Figure 3b.**
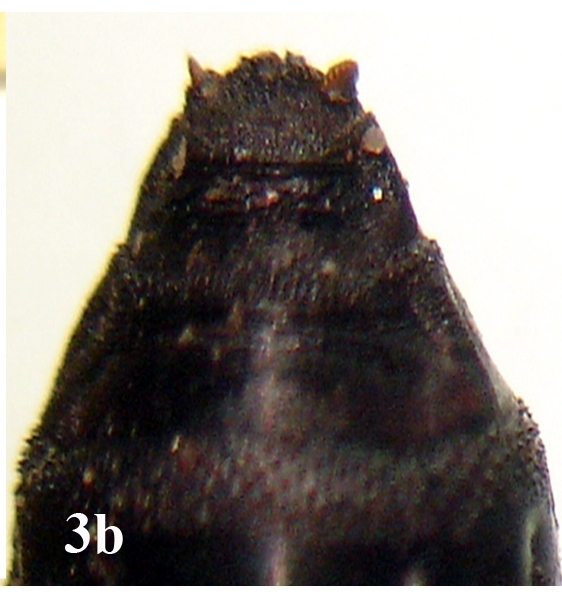
Anterior spiracles

**Figure 3c.**
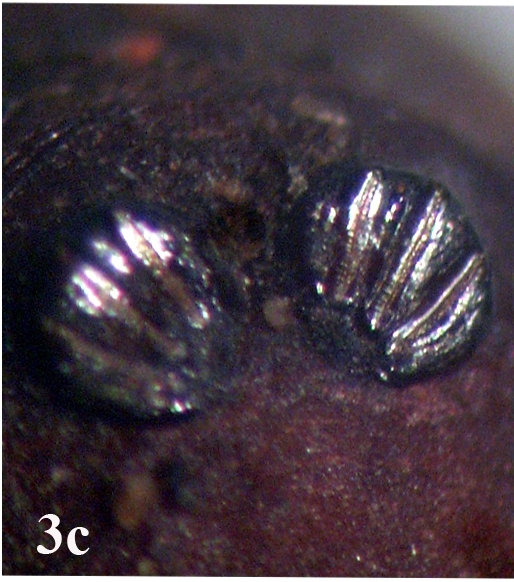
Posterior spiracles

**Figure 4a.**
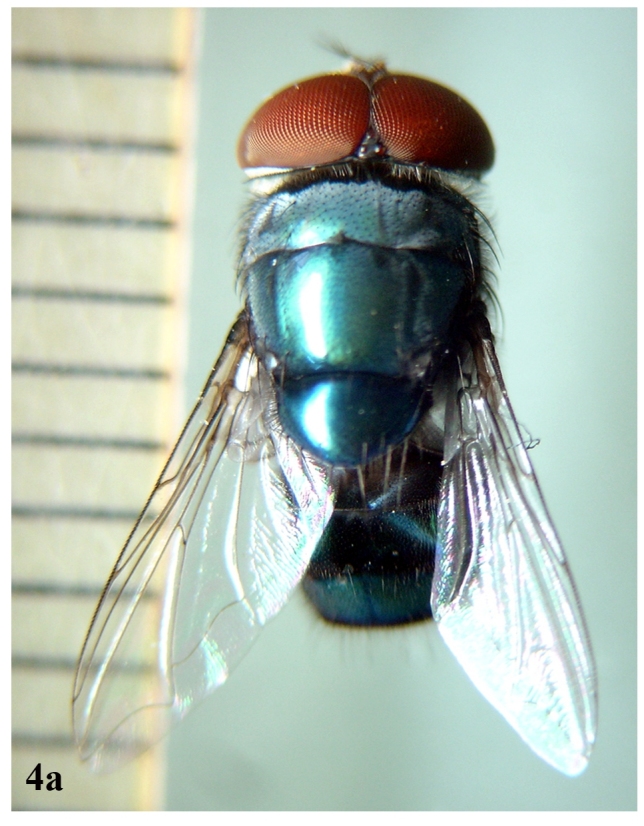
Male dorsal view

**Figure 4b.**
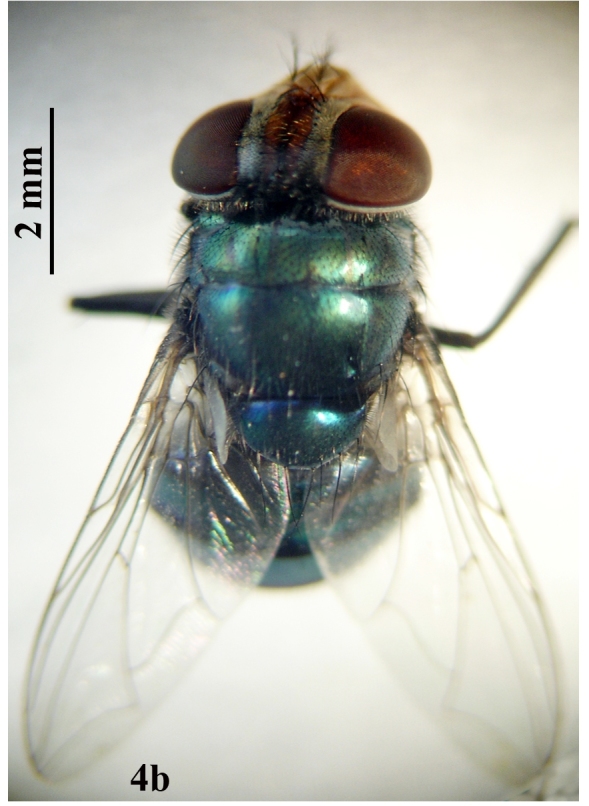
Female dorsal view

**Figure 4c.**
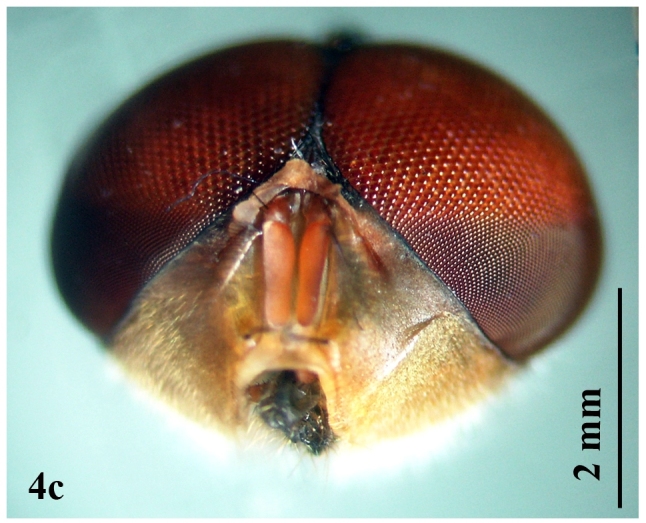
Male head

**Figure 4d.**
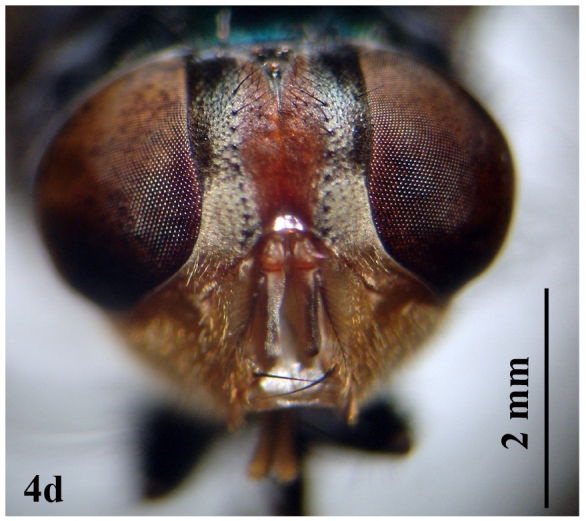
Female head

**Figure 5a.**
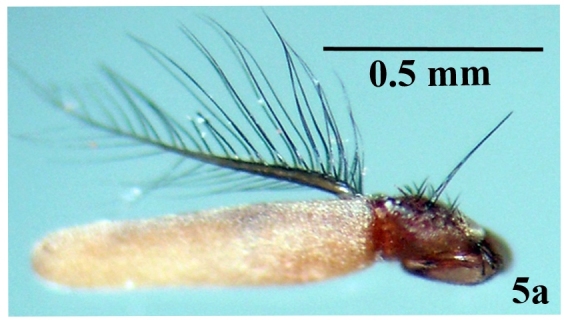
Antennae

**Figure 5b.**
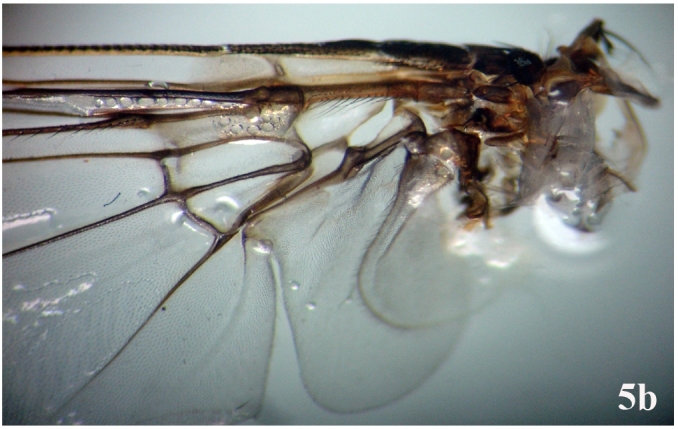
Wing

**Figure 5c.**
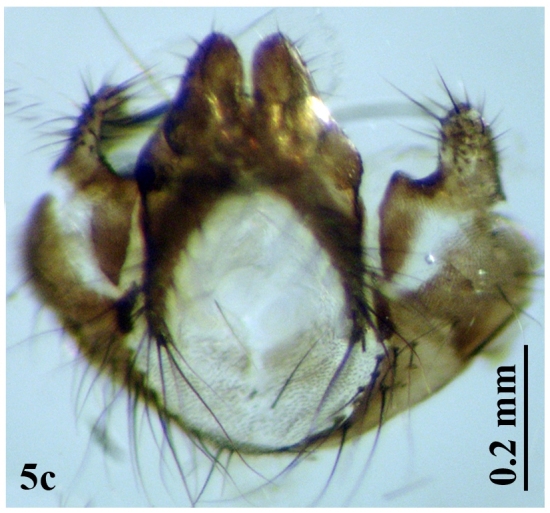
Male genitalia

**Figure 5d.**
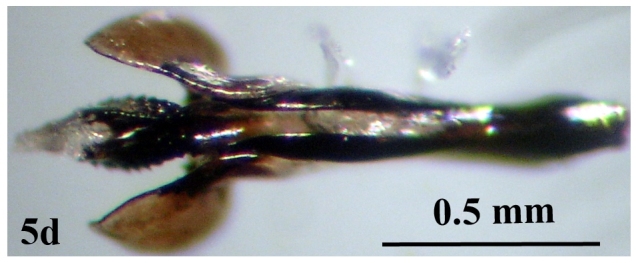
Phallosome
